# Efficacy of atypical antipsychotics in the treatment of fecal incontinence in children and adolescents: a randomized clinical trial

**DOI:** 10.1186/s12887-023-04474-4

**Published:** 2024-01-03

**Authors:** Ghazal Zahed, Somaye Fatahi, Leila Tabatabaee, Negar Imanzadeh, Shaikh Sanjid Seraj, Benjamin Hernández Wolters, Amirhossein Hosseini

**Affiliations:** 1 Department of child and adolescent psychiatry, Shahid Beheshti University of Medical Sciences, Tehran, Iran; 2 Department of Clinical Nutrition & Dietetics, Faculty of Nutrition Sciences and Food Technology, National Nutrition and Food Technology Research Institute, Shahid Beheshti University of Medical Sciences, Tehran, Iran; 3 Pediatric Gastroenterology, Hepatology, and Nutrition Research Center, Research Institute for Children’s Health, Shahid Beheshti University of Medical Sciences, Tehran, Iran; 4 School of pharmacy, Shahid Beheshti University of Medical Sciences, Tehran, Iran; 5 Royal Shrewsbury Hospital, Shrewsbury and Telford NHS Trust, Shrewsbury, Shropshire, SY3 8XQ UK; 6 Health Science Center, Universidad de Guadalajara, Guadalajara, México

**Keywords:** Retentive fecal incontinence, Risperidone, Pediatric, Encopresis, Atypical antipsychotics, Fecal soiling

## Abstract

**Objectives:**

Functional retentive overflow incontinence (retentive FI) is the most common cause of fecal soiling in children. Based on the clinical experiences, the treatment of retentive FI in patients with comorbid psychiatric disorders was accelerated when Risperidone was used as treatment for their psychiatric comorbidities; therefore, this study was conducted to evaluate the effect of risperidone in the treatment of retentive FI in children and adolescents.

**Methods:**

In this double-blind, randomized, placebo-controlled trial, 140 patients aged 4–16 years eligible for the study were randomized into two groups, receiving either 0.25–0.5 mg of Risperidone syrup (n = 70) or maltodextrin syrup (placebo group, n = 70) every 12 h daily for 12 weeks. Sociodemographic data, including age, sex, weight, height, BMI, BMI z-score, and socioeconomic status, was recorded, and the number of nocturnal FI, diurnal FI, and painful defecations was measured.

**Results:**

136 participants (69 on Risperidone and 67 on placebo) were included in the study. Mean age of participants in the intervention and placebo groups were 7.2 ± 2.4 years and 8.0 ± 3.1 years, respectively. The mean number of nocturnal FI (P_trend_=0.39) and diurnal FI (P_trend_=0.48) in patients without psychiatric comorbidities, and the number of painful defecations for participants with and without psychiatric comorbidities (*P* = 0.49, *P* = 0.47, respectively) were not significantly different between the groups, but a significant effect was observed in diurnal FI after Risperidone treatment in patients with psychiatric comorbidities (*P* < 0.001).

**Conclusion:**

Risperidone, when used along with other non-pharmacological interventions, may be helpful in treating FI in pediatric patients with psychiatric comorbidities.

**Supplementary Information:**

The online version contains supplementary material available at 10.1186/s12887-023-04474-4.

## Introduction

According to the DSM-5 (Diagnostic and Statistical Manual of Mental Disorders-Fifth Edition), encopresis is the voluntary or involuntary passage of stool in inappropriate places, such as underwear, in a child with a developmental age of four years or older, once a month or more for at least 3 months, after ruling out organic causes [1]. Fecal incontinence (FI), according to the Rome-IV criteria, is a pediatric gastroenterological problem characterized by recurrent uncontrolled passage of fecal matter into the underwear in children older than 4 years [2]. Depending on pathophysiological mechanisms, FI can be classified into retentive fecal incontinence, which is always associated with constipation and fecal impaction, and non-retentive FI, in which no evidence of constipation or of rectal stools is found [2, 3].

Retentive FI is the most common cause of fecal soiling in children, with a prevalence of 3% and 1.6% of children aged 4 and 10, respectively, and being 3 times more common in males [4]. Patients often recover significantly, with a recovery rate of up to 30–50% in a year and about 50–75% after five years [5]. Retentive FI can lead to embarrassment and guilt in patients making them victims of bullying [6, 7] and is linked to psychiatric disorders in 30 to 50% of pediatric patients, including: attention deficit hyperactivity disorder (ADHD), oppositional defiant disorder (ODD), mood and anxiety disorders, and poor school performance [6, 8–10].

The biopsychosocial nature of fecal incontinence necessitates a comprehensive approach to evaluation and treatment [11]. Thus, commonly used interventions include daily oral administration of laxatives, regular timed intervals on the toilet, and cognitive-behavioral methods to assist children with regular toilet-trained bowels. Family psychoeducational interventions, supportive psychotherapy, and relaxation techniques have been used to alleviate the anxiety surrounding defection. Treatment failure results from chronicity of the FI and from psychiatric comorbidities [12]. Other effective pharmacological treatments for non-retentive FI include imipramine, methylphenidate, and atomoxetine [13–15].

Risperidone, an atypical antipsychotic which acts as a serotoninergic and dopaminergic receptor antagonist, is commonly used to treat a variety of psychiatric disorders in all age groups [16]. Dopamine inhibits colonic movements, gastric emptying, and oro-cecal transit time [17]; hence, it can be hypothesized that dopamine antagonists could be an effective treatment for retentive FI. To our knowledge, no other study has been conducted to examine the effect of Risperidone in patients with fecal incontinence; therefore, the present randomized controlled trial was conducted to evaluate the effect of Risperidone in the treatment of retentive FI in children and adolescents.

## Methods

### Study design

In this double-blind, randomized, placebo-controlled clinical trial, pediatric patients referred to the gastrointestinal clinic of two children’s hospitals in Tehran (Mofid and Loghman) during the period 2021 to 2022 were included. To determine the sample size, sample sizes from studies using other therapeutic interventions (probiotics, interferential (IF) electrical stimulation, pelvic floor muscle (PFM) exercises, and cassia fistula emulsion) [18–20] were used. Roughly 16% of patients reported improvement of FI with probiotic treatment. Thus, a 20% improvement with Risperidone treatment should be expected in this study. Using a 95% significance level with the type I error probability level of 5% (α = 0.05), the type II error probability level of 20% (β = 0.20, power = 80%), and assuming a 10% possible loss, the appropriate sample size was calculated to be 70 subjects in each group, for a total of 140 participants, according to the following formula: n = C x [(P 1(1 - P 1) + P 2(1 - P 2)/ (P 2 – P 1)^2^] [21]. This study was approved by the research ethics committee of Shahid Beheshti University of Medical Sciences, Tehran, Iran (IR.SBMU.MSP.REC.1398.833). This prospective randomized controlled trial has been registered in Iranian Registry of Clinical Trials with IRCT number (IRCT20200203046352N1) at 19/06/2022.

### Patient selection

Patients were included if they fulfilled the following inclusion criteria: demonstrating cooperation after fully understanding the objectives and methods of the study, aged between 4 and 18 years, and diagnosis of retentive FI according to the ROME-IV diagnostic criteria [22], made by the fulfillment of 2 or more of the following criteria for a minimum of 1 month, with insufficient criteria for a diagnosis of irritable bowel syndrome: (1) 2 or fewer defecations in the toilet per week in a child of a developmental age of at least 4 years, (2) at least 1 episode of fecal incontinence per week, (3) history of retentive posturing or excessive volitional stool retention, (4) history of painful or hard bowel movements, (5) presence of a large fecal mass in the rectum, and (6) history of large diameter stools that can obstruct the toilet.

Exclusion criteria consisted of any past medical history of cardiovascular, hepatic, renal, and/or metabolic diseases; morbid obesity; use of medications for psychiatric disorders; pregnant and lactating adolescents; smoking (more than one cigarette in the last week or more than 200 cigarettes in a lifetime); have any previous reported allergies to Risperidone; and prior noncompliance with medication.

#### Informed consent

was obtained from the parents of every included participant. Psychiatric and gastrointestinal assessments were performed by a pediatric psychiatrist using K-SADS [23] and a pediatric gastroenterologist using ROME-IV. The three most common psychiatric disorders in the participants of our study were generalized anxiety disorder (GAD), attention deficit hyperactivity disorder (ADHD), and major depressive disorder (MDD). Sociodemographic data were recorded, including age, sex, weight, height, BMI and BMI z-score (equivalent BMI-for-age percentile), and socioeconomic status.

### Randomization

**Fig. 1:**
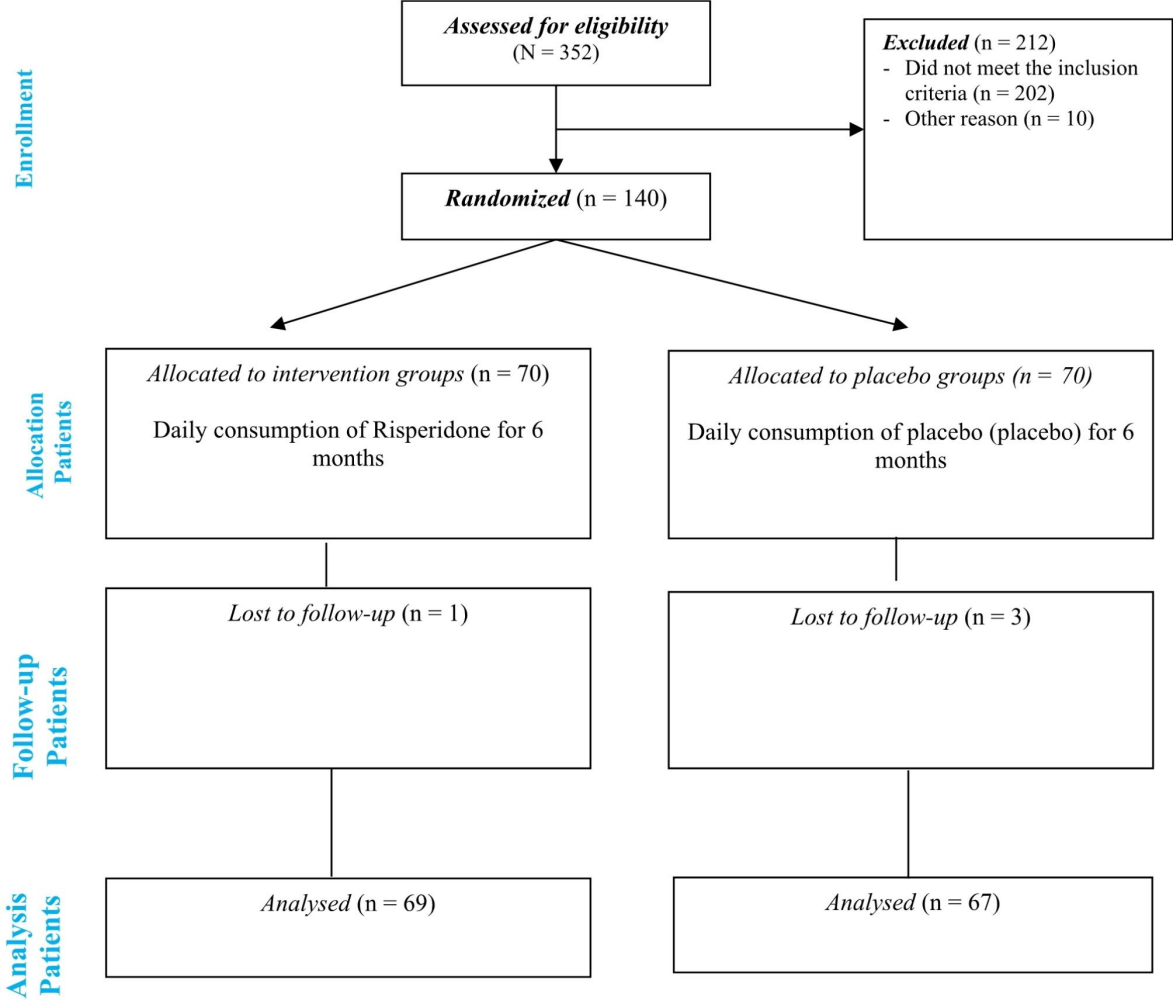
Flow diagram for the trial

140 children and adolescents aged 4–16 met the inclusion criteria and were randomly divided into two groups to receiving either Risperidone or placebo. The flow diagram for the trial is shown in Fig. 1. About half of these patients had newly diagnosed psychiatric disorders (with mild to moderate intensity) and were drug naïve. BMI Z-score and psychiatric disorders can significantly impact the study results, to ensure the uniform distribution of these variables across groups, allocation was randomized using stratified randomization and permuted block randomization methods with quadruple and binary blocks. For all 136 subjects, the quadruple block or double block was obtained using the online site (www.sealedenvelope.com). The company receiving the supplements and placebos inserted these codes on the packages. As each participant entered the study, based on the sequence generated, the drug package in which the code is recorded was delivered to the participant’s parents. The adherence rate of patients during the intervention period was calculated using the following formula: adherence rate = number of packages consumed by the end of the study / number of packages received at the beginning of the study * 100; and patients whose adherence rate was lower than 80% were excluded from the study.

### Interventions (pharmacological and non-pharmacological)

Participants received either 0.25–0.5 mg of Risperidone syrup every 12 h (intervention group) or maltodextrin (placebo group) daily for 12 weeks. To maintain double-blindness within the study, sets of packs containing Risperidone and placebo were prepared by someone other than the researchers and the syrup appearance of placebo and Risperidone were similar. The drugs were given to the parents at the beginning of the study, and then were asked to bring the empty packages after each month (for three months) to ensure compliance with the intervention. In addition to polyethylene glycol, all the participants (two groups) received behavioral intervention and bowel movement habit training (family counseling, education for withholding behaviors and related behavioral interventions, regular toileting, use of stool diaries, and reward systems for successful fecal evacuations) [4]. Non-pharmacological interventions for psychiatric comorbidities, including cognitive behavioral therapy, social skills training, parent management training (PMT), and family therapy were performed by a pediatric mental health professional.

### Statistical analysis

Quantitative variables were reported as mean (standard deviation), and qualitative variables were reported as number of participants (percentage). To compare the mean of the quantitative outcomes between the two groups, independent T-Tests were used to compare the results between baseline and end of the intervention. Chi-square test and Fisher’s exact tests were used to compare qualitative variables between the two groups. The effect of Risperidone on quantitative variables was analyzed by two-way repeated measure ANOVA tests and post-adaptation covariance tests for possible confounding factors. SPSS software version 16 was used for statistical analyses, and a *P*-value < 0.05 was used as significance level for all tests. Intention-to-treat (ITT) analysis was performed as well using the same statistical tests.

## Results

136 participants (69 on Risperidone and 67 on placebo) completed the intervention. Demographic characteristics of each group at baseline are shown in Table 1. Mean age of participants in the intervention and placebo groups were 7.2 ± 2.4 and 8.0 ± 3.1 years, respectively. No statistically significant differences were observed in weight, height, BMI Z-score and BMI (kg/m^2^) among the groups at baseline. The number of girls in each group differed significantly (girls in placebo = 17 (36.2%) and girls in intervention = 30 (63.8%)). 34 subjects in the Risperidone group and 35 in the placebo group had psychiatric comorbidities, with no statistically significant difference between the two groups (*P* = 0.43).

**Table 1:** Baseline characteristics of participants

Variables		All subjects (n = 136)	Risperidone (n = 69)	Placebo (n = 67)	*P* value^a^
Age (yr)		7.6 ± 2.8	7.2 ± 2.4	8.0 ± 3.1	0.10
Sex (girl)		47(34.6)	17(36.2)	30(63.8)	0.02
Weight (kg)		26.9 ± 14.4	27.5 ± 16.0	26.1 ± 12.4	0.66
Height (cm)		121.1 ± 17.1	121.3 ± 16.8	120.7 ± 17.8	0.88
BMI Z-score		-0.11 ± 1.31	0.16 ± 1.30	-0.26 ± 1.31	0.19
BMI (kg/m^2^)		16.3 ± 2.9	16.1 ± 2.5	16.1 ± 2.5	0.56
psychiatric disorders		69(50.7)	34(49.3)	35(50.7)	0.43
SES ^b^					
	strong	66 (48.5)	32(46.3)	34(50.7)	0.78
	weak or moderate	70(51.4)	37(53.6)	33(49.2)	0.65

Data are mean ± SD or n (%).

BMI: body mass index; SES: socioeconomic status.

^a^ Calculated by ANOVA and chi-square tests for quantitative and qualitative variables, respectively.

^b^ Percent

Table 2 shows the gastrointestinal manifestations of participants at baseline. Values for constipation, fissures, hard stool, and compact feces were not significantly different between the groups (*P* > 0.05); however, the presence of hemorrhoids in patients with psychiatric disorders was significantly different between the groups (*P* = 0.025).

**Table 2:** Gastrointestinal manifestations of participants at baseline of trial

variable		All subjects	Placebo (n = 67)	Risperidone (n = 69)	P value ^d^
History of seizures					
Psychiatric disorder	Yes	1(1.4)	1(2.9)	0(0.0)	-
	No	0(0.0)	0(0.0)	0(0.0)	0.52
Thyroid disorder					
Psychiatric disorder	Yes	0(0.0)	0(0.0)	0(0.0)	-
	No	67(100.0)	35(100.0)	32(94.1)	0.39
Abdominal surgery					
Psychiatric disorder	Yes	1(1.4)	1(2.8)	0(0)	0.46
	No	1(1.4)	1(2.8)	0(0)	0.46
Constipation					
Psychiatric disorder	Yes	47(68.1)	22(62.8)	25(73.5)	0.36
	No	43(64.1)	21(60.0)	22(64.7)	0.31
Hemorrhoids					
Psychiatric disorder	Yes	5(7.24)	5(14.2)	0(0.0)	0.025
	No	4(5.97)	3(8.5)	1(2.94)	0.34
Fissure					
Psychiatric disorder	Yes	15(21.7)	8(22.8)	7(20.5)	0.47
	No	11(16.4)	4(11.4)	7(20.5)	0.20
Hard stools					
Psychiatric disorder	Yes	45(65.2)	22(62.8)	23(67.6)	0.56
	No	41(61.1)	19(54.2)	22(64.7)	0.16
Compact feces					
Psychiatric disorder	Yes	43(62.3)	21(60.0)	22(64.7)	0.56
	No	40(59.7)	20(57.1)	20(58.8)	0.46

Data are n (%).

^f^*P* values have been calculated by chi-square tests between groups.

After the intervention, the mean number of nocturnal FI (P_trend_=0.39) and diurnal FI (P_trend_=0.48) in patients without psychiatric comorbidities, and the number of painful defecations for participants with and without psychiatric comorbidities (*P* = 0.49, *P* = 0.47, respectively) were not significantly different between the groups. When comparing post-intervention changes on diurnal and nocturnal FI in patients with concurrent psychiatric comorbidities, a significant effect was observed on diurnal FI after Risperidone treatment (*P* < 0.001), with no significant effect on nocturnal FI (*P* = 0.44) **(**Table 3, Supplementary Figs. 1–3).

**Table 3:** Mean number of stools and percent of painful defecation among participants at baseline and after 3 months

Variable	Baseline		2 Months		3 Months			
	Placebo (n = 67)	Risperidone (n = 69)	Placebo (n = 67)	Risperidone (n = 69)	Placebo (n = 67)	Risperidone (n = 69)	*P* value	*P* value
**Number of nocturnal stools**	0.20(0.95)	0.33(0.65)	0.03(0.21)	0.24(0.58)	0.03(0.17)	0.05(0.22)		0.44^b^
With psychiatric disorder	0.41(1.50)	0.32(0.47)	0.09(0.29)	0.24(0.52)	0.05(0.21)	0.08(0.27)	0.39^a^	
No psychiatric disorder	0.0(0.0)	0.33(0.73)	0.0(0.0)	0.24(0.70)	0.0(0.0)	0.0(0.0)		
**Number of diurnal stools** ^**1**^	5.28(2.73)	6.28(2.79)	4.33(3.35)	3.03(2.92)	1.92(1.81)	1.31(1.83)		< 0.001^b^
With psychiatric disorder	8.24(3.53)	7.01(2.34)	5.42(3.82)	3.75(3.33)	1.80(1.43)	1.50(1.88)	0.48^a^	
No psychiatric disorder	2.31(1.93)	5.55(3.59)	3.23(2.80)	2.31(2.51)	2.05(2.19)	1.13(1.78)		
**Painful defecation** ^**2**^	36(53.7)	46(66.6)	32(47.7)	35(50.7)	7(10.4)	9(13.0)		0.49^c^
With psychiatric disorder	20(29.8)	24(34.7)	10(14.9)	16(23.18)	4(5.9)	5(7.2)	0.49 ^c^	
No psychiatric disorder	16(23.8)	22(31.8)	22(32.8)	19(27.5)	3(4.4)	4(5.7)	0.47 ^c^	

^a^*P* values calculated by Repeated measures for time*group and adjusted for sex, socioeconomic status and significant values of Table 2

^b^*P* values calculated by Repeated measures for time* psychiatric disorder and adjusted for sex, socioeconomic status and significant values of Table 2

^c^*P* values calculated by chi-square tests

^1^ Data are mean ± SD.

^2^ Data are n (%).

## Discussion

A high prevalence of psychiatric comorbidities, especially ADHD and ODD, has been reported in pediatric patients with FI [6, 8, 9], with comorbid internalizing and externalizing psychiatric disorders considered predictors of poor outcome [24, 25]. Based on clinical experience, educational, psychological, and behavioral interventions have been effective for patients with FI and comorbid psychiatric disorders, while psychopharmacologic interventions have been modestly effective as part of this multidisciplinary treatment program [6, 13–15, 26]. Atypical antipsychotics are commonly prescribed for various psychiatric disorders, such as psychotic, mood, anxiety, tics, disruptive behavior, and obsessive-compulsive disorders [27]. To our knowledge, this is the first double-blind, randomized, placebo-controlled trial of Risperidone for treatment of retentive FI in a clinical setting.

According to the results of this study, in the presence of psychiatric comorbidities, Risperidone supplemented other traditional interventions to significantly reduce the frequency of diurnal FI (*P* < 0.001), with no significant impact on the frequency of nocturnal FI. In contrast with these results, very few case reports of Risperidone-induced fecal incontinence have been reported in the literature. For example, Herguner and Mukaddes [28] described double incontinence (fecal plus urinary incontinence) after 1 to 3 weeks of initiation of Risperidone treatment in two patients with autistic disorder; however, the reported side effects were not severe. In addition, there are several other case reports associating the use of Risperidone with urinary and fecal incontinence in cases of autistic spectrum disorder (ASD) [29], attention deficit hyperactivity disorder, and ODD [30]. In one of these studies, the researchers stated that the probability of such a complication is of 7 based on the Naranjo Adverse Drug Reaction Probability Scale, being graded as “probable” according to the scale’s definition [30]. Since retention behavior is central to the maintenance of constipation, fecal impaction, and retentive FI, the improvement in these factors in patients with psychiatric disorders could be associated with psychological and behavioral improvement and a change in body perception. Risperidone has a high affinity for dopamine D2, serotonin 5-HT2A, adrenergic α1 and α2, and histamine H1 receptors, and a moderate affinity for serotonin 5-HT1C, 5-HT1D, and 5-HT2A receptors [27]. It is not clear whether the mechanism of action of Risperidone on retentive FI is central or peripheral, but it is thought that, since dopamine inhibits colonic movements and prolongs gastrointestinal transit time [17, 31], the antagonistic effect of Risperidone on dopamine may be the cause of the improvement in retentive FI [27].

In the current literature, the relationship between atypical antipsychotics and FI is evident. In a systematic review conducted in 2021, Arasteh et al. pointed out that atypical antipsychotic drugs can cause FI, which may be due to α1-adrenergic blockade, sedative effects, or blockage of the pudendal reflexes [32]. The same study demonstrated that the most common adverse effects of Risperidone were weight gain and mild sedation, while the most common drug-related reasons for discontinuing Risperidone are extrapyramidal manifestations. However, among the atypical antipsychotic drugs, Risperidone is one of the least sedative [33].

Previous studies have suggested that Imipramine, Methylphenidate, and Atomoxetine may help treat FI [13–15]. Imipramine is a Tricyclic Antidepressant (TCA) with an anticholinergic effect similar to Loperamide, reducing gastrointestinal motility and increasing sphincter tone. Some studies indicate that Imipramine can be helpful in treating non-retentive FI, but due to cardiovascular adverse effects, TCAs should not be routinely prescribed [6, 14]. In a 2012 study, Huang and Chien showed that inhibition of gastric emptying and intestinal transit induced by amphetamine is due to an effect on the dopaminergic system (via D1 and D2 receptors) and, to some extent, adrenergic receptors. In a 2013 case report, Yılmaz and Akça noted that Methylphenidate lead to recovery from FI [15]. Furthermore, Golubchik and Weizman, in 2013, pointed out that the direct impact of Methylphenidate, Imipramine, and Atomoxetine on self-organizing skills, impulse control, and executive functioning may enable children to recognize and respond to internal cues for defecation, making them useful as part of the treatment process for FI [15, 26]. The effects of Atomoxetine in the treatment of FI have been reported in the literature; however, one of the side effects of Atomoxetine, like Risperidone, is constipation, and thus, administration of Atomoxetine to patients with ADHD and comorbid FI can either improve or worsen FI [13, 27, 34, 35]. These findings may shed a light on the effects of central nervous system medications on the gastrointestinal tract [36].

To our knowledge, this is the first study investigating the effect of Risperidone on FI. The adequate sensitivity of the Rome IV Diagnostic Questionnaire [37] for identifying patients with FI improves the accuracy and correctness of our study. However, our study has limitations that serve as perspectives. First, we did not conduct precise objective assessments of symptom severity and improvement by validated quality of life instruments and fecal incontinence questionnaires (e.g.: Wexner score), which are commonly used for adults and can provide more reliability for the evaluation of such interventions. Second, we could not properly examine the participants’ eating habits as one of the factors affecting fecal incontinence and constipation; therefore, these results were not mentioned in this study. Third, adverse effects such as dizziness, somnolence, nausea, hyperprolactinemia, weight gain (more common in children than adults), extrapyramidal symptoms (highly dose-dependent), tardive and withdrawal dyskinesia, daytime hypersomnia, anxiety, nausea and vomiting, diabetes mellitus, hyperlipidemia, anxiety, rhinitis, and prolonged QTc may occur in some people following the use of Risperidone [38, 39]. According to previous studies, Risperidone is prescribed for a few weeks up to 4 months and no more than 1-1.5 mg/day for the treatment of psychiatric disorders in children and adolescents, except in severe and chronic cases such as schizophrenia and chronic mood disorders [40, 41]. Thus, although a safe dose was used for the intervention, we could not correctly identify the possible side effects of the intervention with this drug on the participants, perhaps due to the short duration of the intervention. Four, the male predominance and gender imbalance between the treatment and placebo groups may question the generalizability of the results, which was a result of limited resources and lack of stratification in our study design. Finally, given the high comorbidity of psychiatric disorders with FI, it may be necessary to develop a comprehensive interdisciplinary treatment plan, taking the possible benefits of psychopharmacotherapy into account.

## Conclusion

Based on our findings, Risperidone, commonly prescribed for psychiatric disorders, may be helpful in the treatment of FI in pediatric patients with psychiatric comorbidities when used along with other non-pharmacological interventions, although no benefit was observed in patients without psychiatric comorbidities. Future studies should be conducted in a multicenter setting with a larger sample size to provide further evidence on the effects of Risperidone on FI.

### Electronic supplementary material

Below is the link to the electronic supplementary material.

Supplementary Material 1

## Data Availability

Access to the data of this study can be obtained through the corresponding author.
