# Supplementary material for: Efficacy of atypical antipsychotics in the treatment of fecal incontinence in children and adolescents: a randomized clinical trial
Source: BMC Pediatr. 2024 Jan 3;24:7. doi: 10.1186/s12887-023-04474-4 (PMC10763209; doi:10.1186/s12887-023-04474-4)
Supplement: Supplementary file 1 — Supplementary Material 1 [file 12887_2023_4474_MOESM1_ESM.pdf]

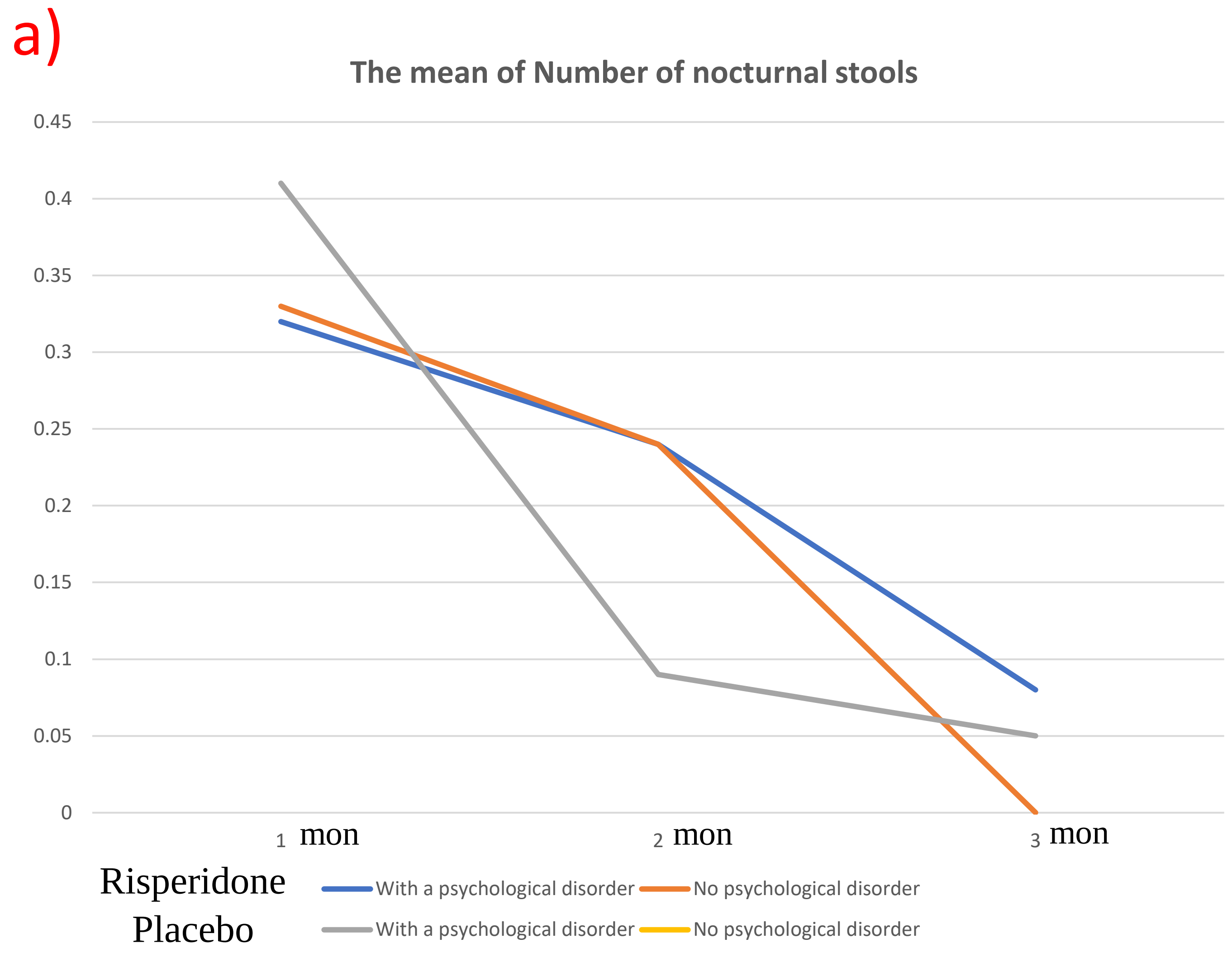

a)
The mean of Number of nocturnal stools
0.45
0.4
0.35
0.3
0.25
0.2
0.15
0.1
0.05
0
1 mon
2 mon
3 mon
Risperidone
Placebo
With a psychological disorder
No psychological disorder
With a psychological disorder
No psychological disorder

b)

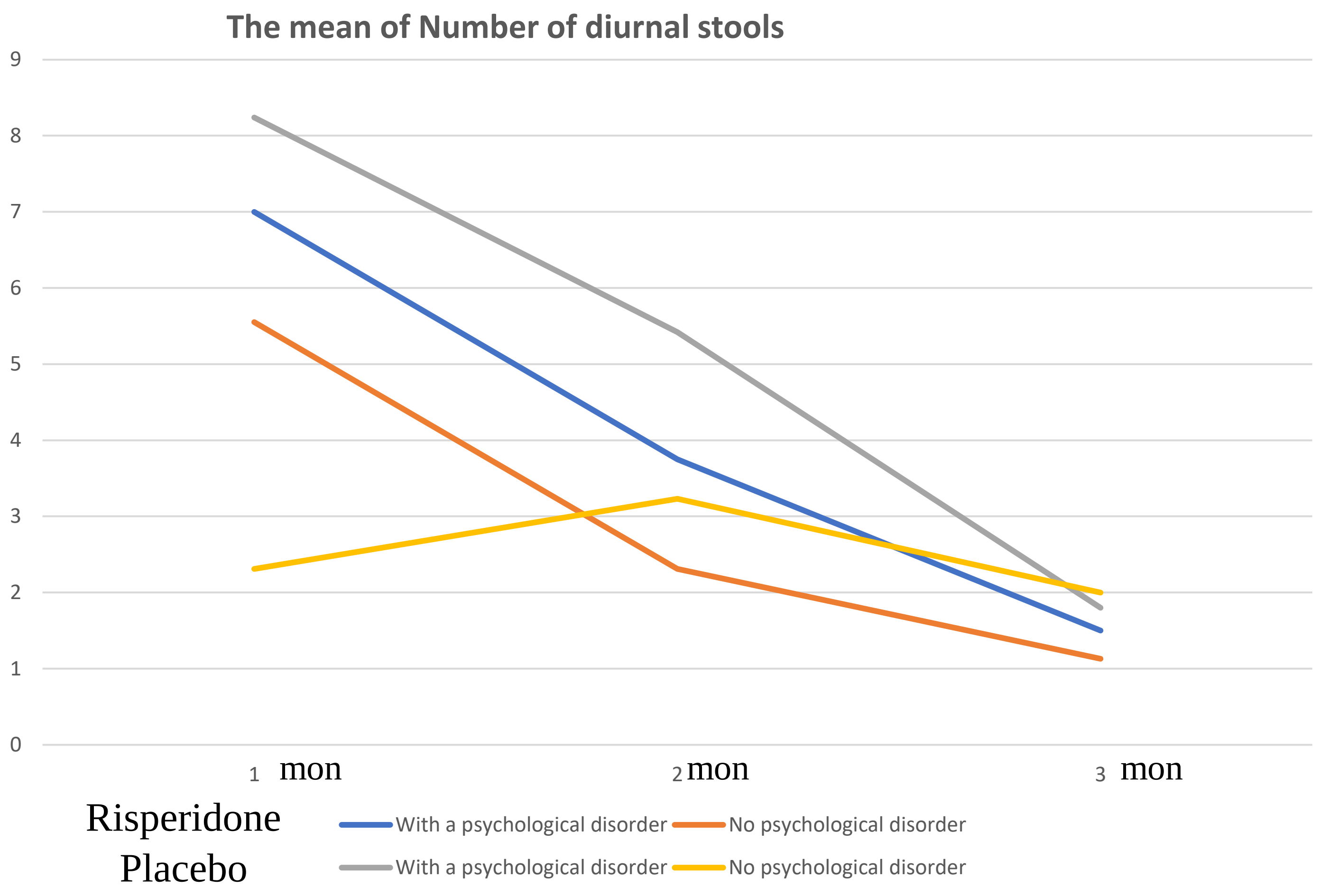

The mean of Number of diurnal stools
9
8
7
6
5
4
3
2
1
0
1 mon
2 mon
3 mon
Risperidone
Placebo
With a psychological disorder
No psychological disorder
With a psychological disorder
No psychological disorder

c)

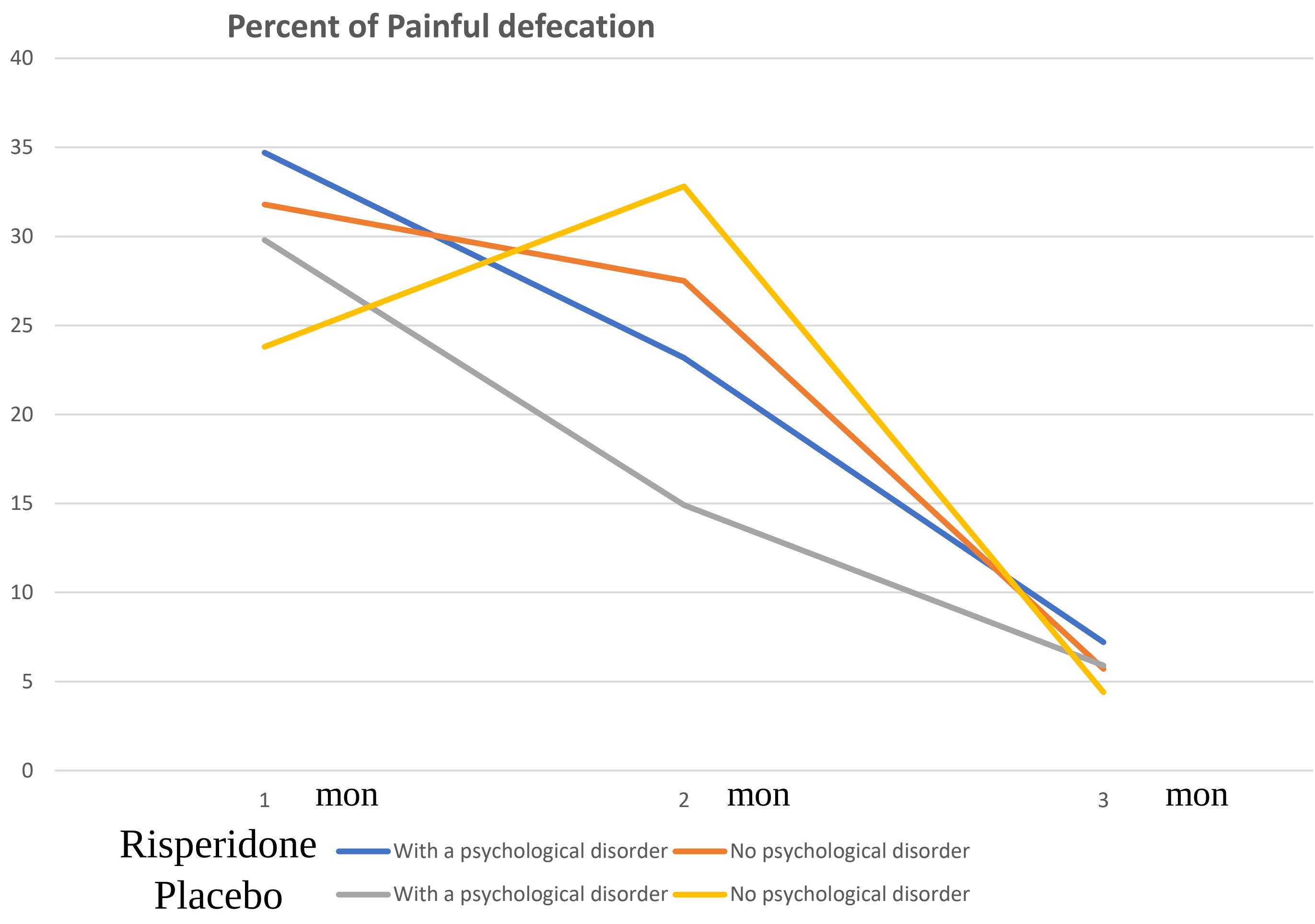

Supplementary Figures 1. Mean number of a) Number of nocturnal stools, b) Number of diurnal stools and c) percent of Painful defecation among participants at baseline and after 3 months
